# Biotransformation of the Mycotoxin Deoxynivalenol in Fusarium Resistant and Susceptible Near Isogenic Wheat Lines

**DOI:** 10.1371/journal.pone.0119656

**Published:** 2015-03-16

**Authors:** Bernhard Kluger, Christoph Bueschl, Marc Lemmens, Herbert Michlmayr, Alexandra Malachova, Andrea Koutnik, Imer Maloku, Franz Berthiller, Gerhard Adam, Rudolf Krska, Rainer Schuhmacher

**Affiliations:** 1 Center for Analytical Chemistry, Department for Agrobiotechnology (IFA-Tulln), University of Natural Resources and Life Sciences, Vienna (BOKU), Tulln, Austria; 2 Institute for Biotechnology in Plant Production, Department for Agrobiotechnology (IFA-Tulln), University of Natural Resources and Life Sciences, Vienna (BOKU), Tulln, Austria; 3 Department of Applied Genetics and Cell Biology, University of Natural Resources and Life Sciences, Vienna (BOKU), Tulln, Austria; USDA, UNITED STATES

## Abstract

In this study, a total of nine different biotransformation products of the *Fusarium mycotoxin* deoxynivalenol (DON) formed in wheat during detoxification of the toxin are characterized by liquid chromatography—high resolution mass spectrometry (LC-HRMS). The detected metabolites suggest that DON is conjugated to endogenous metabolites via two major metabolism routes, namely 1) glucosylation (DON-3-glucoside, DON-di-hexoside, 15-acetyl-DON-3-glucoside, DON-malonylglucoside) and 2) glutathione conjugation (DON-S-glutathione, “DON-2H”-S-glutathione, DON-S-cysteinyl-glycine and DON-S-cysteine). Furthermore, conjugation of DON to a putative sugar alcohol (hexitol) was found. A molar mass balance for the cultivar ‘Remus’ treated with 1 mg DON revealed that under the test conditions approximately 15% of the added DON were transformed into DON-3-glucoside and another 19% were transformed to the remaining eight biotransformation products or irreversibly bound to the plant matrix. Additionally, metabolite abundance was monitored as a function of time for each DON derivative and was established for six DON treated wheat lines (1 mg/ear) differing in resistance quantitative trait loci (QTL) *Fhb1* and/or *Qfhs.ifa-5A*. All cultivars carrying QTL *Fhb1* showed similar metabolism kinetics: Formation of DON-Glc was faster, while DON-GSH production was less efficient compared to cultivars which lacked the resistance QTL *Fhb1*. Moreover, all wheat lines harboring *Fhb1* showed significantly elevated D3G/DON abundance ratios.

## Introduction

An effective response to stress conditions is crucial for sessile organisms such as plants which are exposed to external influences from the environment. In this respect, plants have developed a multitude of mechanisms to cope with abiotic (e.g. high intensity UV light, temperature changes, drought, salinity, xenobiotics) [1] as well as biotic stresses most commonly caused by microorganisms such as bacteria and filamentous fungi. Whereas biotrophic fungi depend on the living host, fungal pathogens with a predominantly necrotrophic lifestyle eventually kill their host cells by secreting toxic secondary metabolites and grow on the necrotic plant tissue [2].

Fusarium Head Blight (FHB, scab) is caused by various *Fusarium* species and occurs on wheat, and other small-grain cereals. It is responsible for severe yield and quality losses and considered to be a relevant global hazard for food safety and security [3]. Wheat, barley and other small grain cereals are affected by *Fusarium graminearum*, one of the most important species causing FHB [4]. The *F*. *graminearum* derived mycotoxin deoxynivalenol (DON) belongs to the group of trichothecene mycotoxins and plays a key role in FHB. DON acts as a virulence factor by inhibiting protein biosynthesis of the host [5]. As a consequence of fungal infection, the mycotoxin is a frequent contaminant of food and feed and has a great impact on both human and animal health [6]. Mechanisms of action and toxicological relevance of the toxin has been reviewed by Pestka in 2010 [7].

Mechanisms to counteract microbial toxins and other xenobiotics include their detoxification by conjugation to endogenous metabolites (e.g. by glucosylation, acylation, conjugation to amino acids and to the dedicated peptide glutathione). The resulting biotransformation products are subsequently transported to the vacuole or to the apoplast. Moreover, the toxin conjugates can be further incorporated into cell wall components [8]. So far, most knowledge about detoxification mechanisms of xenobiotics has been generated by metabolism studies of pesticides [9–12]. Despite the importance for fungal virulence and plant disease resistance, limited information exists regarding detoxification of mycotoxins *in planta*. Several reviews give an overview of the plant mediated metabolism involved in the detoxification of mycotoxins and the resulting conjugates [8,13,14]. These modified mycotoxins have an altered structure and different mass, making them difficult to detect by conventional analytical approaches and they have the potential to be reactivated to the parental toxin (e.g. by hydrolyzation in the intestinal tract [15,16]) and are therefore often designated to be masked mycotoxins. According to a recent proposal, the term masked mycotoxin should only be used for plant metabolites [17]. The difficulties in detection of conjugated mycotoxins lead to an underestimation of total mycotoxin content in contaminated food and feed [18]. With respect to DON, the probably most important detoxification reaction to reduce the toxicity of DON *in planta* is its conjugation to glucose first reported in *Arabidopsis thaliana* [19], and also reported for naturally *F*. *graminearum* inoculated and contaminated wheat [20]. Additionally, the occurrence of DON oligoglucosides such as DON-di-glucoside, DON-tri-glucoside and DON-tetra-glucoside in malt, beer and bread has been reported previously [21].

For detection of mycotoxins and their derived biotransformation products, liquid chromatography (LC) combined with mass spectrometry (MS) is currently one of the most powerful techniques due to its high selectivity and sensitivity. The concept of using isotopic patterns to study the metabolism of labeled tracers have long been known and has the advantage that the detected biotransformation products can be linked to the studied tracer substance [22]. Recently developed novel stable isotope labeling assisted approaches enable the automated, untargeted profiling of biotransformation products of xenobiotics, including mycotoxins *in planta* [23]. Kluger *et al*., reported the detection of novel GSH related conjugation products of DON in wheat such as DON-glutathione (GSH) and its corresponding degradation products DON-S-cysteinylglycine (DON-S-cys-gly) and DON-S-cysteine (DON-S-cys) [24]. Besides its putative relevance as masked mycotoxins, contributing to the overall toxicity, the formation of conjugated mycotoxins may add to the current understanding of resistance mechanisms in host plants.

FHB resistance in wheat has been associated with more than 100 quantitative trait loci (QTL), with only few QTL genetically mediating FHB resistance [25], among them the most important *Fhb1* (formerly known as *Qfhs.ndsu-3BS*) [26] and *Qfhs.ifa-5A* [27]. *Fhb1* has been attributed to a more efficient glucosylation of DON to DON-3*-β-D*-glucoside (D3G). The gene mediating *Fhb1* resistance is still unknown. Lemmens *et al*. [28] reported that wheat lines harboring *Fhb1* showed an increased resistance to the phytotoxic effect of DON applied to flowering wheat ears. This increased resistance was associated with an increased D3G/DON concentration ratio in DON treated wheat kernels harvested after full ripening. Based on this observation, the mechanism underlying *Fhb1* based resistance was postulated to be attributed to either a gene encoding a UDP-glucosyltransferase (UGT) or exhibiting a regulatory function with respect to D3G formation. In a more recent metabolomics study Gunnaiah *et al*., [29] also investigated the mode of action of the *Fhb1* QTL in wheat lines. Flowering wheat ears were infected with spores of *F*. *graminearum* and treated spikelets were harvested 72 hours after infection. In contrast to the findings of Lemmens *et al*., measurements of DON and D3G content revealed that neither the DON content nor D3G/DON ratio in inoculated spikelets were affected by the presence of *Fhb1* under the tested conditions. The authors concluded that *Fhb1* is not related to DON glucosylation and from further metabolomics data they proposed that the formation of phenylpropanoid amides (PPA) was increased in *Fhb1* containing wheat lines. Thus, Gunnaiah *et al*. [29], concluded that PPAs serve as cell wall constituents of rachis tissue and *Fhb1* is confering FHB resistance by processes resulting in cell wall thickening, which leads to physical barriers and in turn prevents fungal growth from the infected spikelet into the rachis.

The study presented here is carried out in continuation of previously published work [24] that describes an untargeted stable isotope labeling assisted LC-HRMS based screening approach, which resulted in the detection of nine different DON biotransformation products in wheat. Here we present the tentative annotation of the remaining biotransformation products and show comparative metabolic kinetics of six different wheat lines: the resistant wheat variety ‘CM-82036’ harboring *Fhb1* and *Qfhs.ifa-5A*, the susceptible variety ‘Remus’ and 4 near isogenic lines (98% homology to ‘Remus’ with all possible combinations of both QTL) after treatment with DON.

## Materials and Methods

### Chemicals and reagents

Acetonitrile (ACN, HiPerSolvChromanorm, HPLC gradient grade) was obtained from VWR (Vienna, Austria); methanol (MeOH, LiChrosolv, LC gradient grade) was purchased from Merck (Darmstadt, Germany); formic acid (FA, MS grade) was obtained from Sigma-Aldrich (Vienna, Austria). Water was purified successively by reverse osmosis and an ELGA Purelab Ultra-AN-MK2 system (Veolia Water, Vienna, Austria). Deoxynivalenol (DON) standard for LC-HRMS analysis was obtained from Romer Labs GmbH (Tulln, Austria) as a stock solution of 100 mg L^-1^ in ACN. Stock solution of deoxynivalenol-3-*β-D*-glucoside (D3G) at a concentration of 1.61 g L^-1^ was prepared as described by Berthiller *et al*., [20].

### Wheat lines and cultivation of wheat plants

For this study six different spring wheat (*Triticum aestivum* L.) lines were used. The resistant parent ‘CM-82036–1TP-10Y-OST-10Y-OM-OFC’ (abbreviated to ‘CM-82036’) has a very high level of resistance against FHB and against DON [28]. The second parent ‘Remus’ is a spring wheat cultivar highly susceptible to FHB and DON. Moreover, four different near isogenic lines (NILs) were used, which had been developed from one BC5F1 plant with ‘Remus’ as recurrent parent (5 backcrosses), which differed in two validated QTLs related to the FHB resistance level (*Fhb1* and *Qfhs.ifa-5A*) [30]. The NILs C1–C4 used in this study contain different combinations of the resistance alleles. C1 (+*Fhb1*/+ *Qfhs.ifa-5A*), C2 (+*Fhb1*), C3 (+*Qfhs.ifa-5A*) and C4 (none) were cultivated in parallel with both parent lines as described below.

Seeds of the spring wheat lines were germinated and seedlings were planted in pots (diameter 23 cm) with homemade soil (mix of 500 L heat-sterilized compost, 250 L peat, 10 kg sand and 250 g rock flour) using a completely randomized block with five biological replications. Plants were grown in a greenhouse under environmentally controlled settings for light, temperature and relative air humidity. For a detailed description of the growth conditions during plant development, the reader is referred to the publication of Warth and co-workers [31]. From the start of DON application until the end of the experiment including sampling, the plants were illuminated for 16 hours/day and the temperature was set at 20°C during illumination and at 18°C at night. At the onset of anthesis each cultivar was treated using an aqueous DON solution as described in detail below.

### DON- and mock treatment of wheat plants

At the beginning of anthesis plants of each cultivar (‘CM-82036’, C1, C2, C3, C4 and ‘Remus’) were treated either with an aqueous DON solution (5 g L^-1^) or with water (mock) according to the following procedure: at time point zero 10 μL DON solution were injected in each of two adjacent spikelets in the lower part of a flowering ear. In total 20 spikelets were treated, by repeating the treatment with spikelets located above those treated before resulting in a total amount of 1 mg DON/wheat ear. 0, 12, 24, 48 and 96h post treatment, inoculated spikelets were sampled and immediately shock-frozen in liquid nitrogen (n = 5 replicates per treatment and time point) resulting in a total of 50 samples for each wheat line.

In order to establish a molar mass balance for DON and D3G, 10 wheat ears of the cultivar ‘Remus’ were inoculated with a total of 1 mg DON at anthesis using the same procedure as mentioned above. Treated spikelets were harvested after 0 hours and 96 hours after treatment (n = 5 wheat ears per time point), immediately immersed in liquid nitrogen and extracted by the same procedure as all other samples.

### Sample preparation for LC-HRMS measurements

All wheat samples were milled in frozen conditions separately for 2 min at 30 Hz to give a fine powder by use of a ball mill (MM 301 Retsch, Haan, Germany) with pre-cooled (liquid nitrogen) 50 mL-stainless-steel grinding jars (Retsch) and a ø 25 mm-stainless steel ball (Retsch). 100 ± 5 mg of homogenized, frozen plant material were weighed into 1.5 mL-Eppendorf tubes. Extraction was performed by adding 1 mL of pre-cooled MeOH:water 75/25 (v/v) including 0.1% formic acid, vortexing for 10 s, and subsequent treatment in an ultrasonic bath (frequency 47 kHz, power: 105 W) at room temperature for 15 min [32]. Samples were centrifuged for 10 min at 19,000 g at 4°C.

An aliquot of the supernatant was transferred to another 1.5 ml-Eppendorf tube and pre-cooled water containing 0.1% formic acid was added to achieve a final MeOH:water ratio of 1:1 (v/v). Finally, the samples were vortexed for 10 s before transfer into HPLC vials for LC-HRMS measurements.

### Preparation of standards

Monoglucosides of the DON-derivatives 15-Acetyl-DON (15-ADON) and 3,7,8,15-tetrahydroscirpene (THS) [33] were produced with a recently identified DON-specific glucosyltransferase [34]. The protein was expressed with the plasmid pKLD116 (N-terminal His6-tag and maltose binding tag in tandem; [35]) using *Escherichia coli* Rosetta (DE3) (Novagen, Madison, WI) as expression host. Protein purification was performed on a 5 mL His Trap FF Column (GE Healthcare, Vienna Austria) following the suppliers instructions. Glucosylation was performed using 1 mg mL^-1^ purified glucosyltransferase and 100 mM Tris/Cl (pH 7) at 37°C for an incubation time of 24 h. UDP-glucose was added to the substrate resulting in 1.5 molar excess.

### LC-HRMS(/MS) analysis

#### Screening for DON and its biotransformation products.

LC-HRMS full scan measurements were carried out as described earlier [36] using the following instrumentation. A UHPLC system (Accela, Thermo Fisher Scientific, San Jose, CA, USA) coupled to a LTQ Orbitrap XL (Thermo Fisher Scientific) equipped with an electrospray ionization (ESI) source. Xcalibur 2.1.0 software was used to control the mass spectrometer and record the data. For HPLC separation, a reversed-phase XBridge C_18_, 150 x 2.1 mm i.d., 3.5 μm particle size (Waters, Dublin, Ireland) analytical column was used at a flow rate of 250 μL min^-1^ at 25°C in a gradient program (injection volume 10 μL). Eluent A was water, eluent B was MeOH, both containing 0.1% formic acid (v/v). The chromatographic method held the initial mobile phase composition (10% B) constant for 2 min, followed by a linear gradient to 100% B within 30 min. This final condition was held for 5 min, followed by 8 min of column re-equilibration at 10% B. The ESI interface was operated in positive ion mode at 4 kV. The Orbitrap mass analyzer was operated in full scan mode with a scan range of *m/z* 100–1000 and a resolving power setting of 60,000 FWHM at *m/z* 400. Samples of individual wheat lines plus quality control samples were measured in separate sequences resulting in a total of six measurement sequences. Qualitative analysis of LC-HRMS data was performed using Xcalibur 2.1.0 QualBrowser software. For relative and absolute quantification of DON and its biotransformation products Thermo Xcalibur 2.1.0 QuanBrowser software was employed. All automatically integrated peak areas were inspected manually, and corrected if necessary.

For structure characterization of DON derivatives, LC-HRMS data files were inspected manually for the presence of DON and its nine biotransformation products, which had been formerly found in an untargeted profiling study [24]. Extracted ion chromatograms (EICs) of each biotransformation product were checked for chromatographic peak shape, retention time similarity of less than ± 0.2 min and mass deviation of less than ± 3 ppm from the exact mass of the proposed biotransformation product.

#### Structure annotation of DON conjugates.

LC-HRMS/MS product ion spectra of DON biotransformation products were recorded by using the same HPLC gradient program. Fragmentation was performed in the collision induced dissociation (CID) mode and subsequent fragment detection in the Orbitrap with a resolving power setting of 7,500 FWHM (at *m/z* 400) and an isolation width setting for the precursor of 3 u. All LC-HRMS/MS spectra were recorded in centroid mode, while *m/z* range and relative collision energy were adjusted to the respective precursors of DON biotransformation products.

#### Absolute quantitation of DON and D3G for establishment of mass balance.

For calibration, DON and D3G stock solutions were prepared separately at different levels and aliquots (15 μL) were spiked into 135 μL of an untreated ‘Remus’ sample extract resulting in concentration levels of 0, 1, 3, 5, 7 and 10 mg L^-1^. Samples for the molar mass balance for DON and D3G were prepared exactly as mentioned above but were further diluted 1:10 (v/v) with water containing 0.1% aqueous formic acid. In parallel the sample extract of the untreated ‘Remus’ wheat ear was diluted 1:10 (v/v) with water containing 0.1% aqueous formic acid.

#### Relative quantification of DON and its biotransformation products.

For relative quantification, data were normalized to facilitate a comparison of concentration levels across all six wheat lines. Therefore, within every measurement sequence the integrated peak area of the respective DON derivatives and for each experimental sample was divided by the average peak area of the respective metabolite in the aggregate control sample (preparation is described in the section “Quality control sample for LC-HRMS analysis”) in the same measurement sequence. Finally, the peak area ratios of each experimental sample were normalized to the highest value found across all of the tested wheat lines (except for DON, where each time course was normalized separately for each cultivar). Average value and standard deviation of the normalized peak area ratio were calculated for each time point (n = 5) and for every wheat line, the resulting area ratios for each biotransformation product were plotted over time.

#### Univariate Statistical Analysis.

Time series plots were created with Python 2.6 (https://www.python.org/; last accessed 5. Aug. 2014) and matplotlib (v. 1.3; [37]). Significance testing (two-sided, non-paired t-test with 5% significance threshold) between the groups of wheat genotypes harboring QTL *Fhb1* (CM, C1, C2) or not (C3, C4, Remus) was performed with R (v. 2.15.2; [38]. A p-value of less than 5% is indicated with a ‘*’ character.

#### Quality control sample for LC-HRMS analysis.

For quality control purposes, one aggregate control sample was produced and measured in regular intervals in parallel to each wheat line in order to compensate for variations during sample preparation and drifts of the mass spectrometer. To this end, aliquots of milled experimental wheat ears representing all six wheat lines, treatments (DON, mock) and time points (0, 12, 24, 48, 96h) were pooled together and milled again in the Retsch mill for homogenization. During preparation of experimental samples, control samples were prepared in parallel and measured at regular intervals throughout the respective LC-HRMS sequence. Furthermore, reference standards dissolved in pure solvent were included in each sequence to monitor mass precision, sensitivity and retention time drifts of the LC-HRMS measurement step.

## Results and Discussion

### Screening for DON and its biotransformation products

Previously, D3G [39] and DON-di-glucoside [21] have been described as wheat derived DON metabolites. In a more recent study, a novel screening strategy for the studying of metabolites derived from exogenous tracers using stable isotopic labeling has been presented. The screening approach was exemplified with the application of a 1+1 (v/v) mixture of native DON and U-^13^C labeled DON to flowering wheat plants of the cultivar Remus as described previously [24]. Further detailed data evaluation of that study revealed a total of nine different DON derivatives, all containing the DON moiety with all 15 carbon atoms. These toxin derivatives, including the already published D3G, DON-GSH and two of its further degradation products (putatively identified on the basis of accurate mass and LC-HRMS/MS product ion spectra) served as candidates for a targeted screening in native DON treated wheat plants as described in this work. Based on LC-HRMS measurements using tolerance limits of ± 3 ppm for mass deviation compared to the exact mass postulated and ± 0.2 min for retention time similarity, the same nine DON biotransformation products were also observed in DON treated wheat samples in this investigation (see Table 1). DON and two of its biotransformation products have been identified based on authentic reference standards (marked with ‘**’ in Table 1), while further five biotransformation products were additionally putatively identified based on accurate *m/z* values and LC-HRMS/MS spectra (marked with ‘*’ in Table 1). Evaluation of the isotopic fine structure revealed the presence of sulfur in four of the nine DON biotransformation products.

**Table 1:** Overview of nine different DON biotransformation products in DON treated wheat samples. Initial annotation for all biotransformation products was performed based on the maximum mass deviation of ± 3 ppm and relative retention order (deviation retention time: ± 0.2 min) compared to the same biotransformation products annotated in the former study [24].

#	Name	RT (min)	Ion species	Theoretic mass (*m/z*)	Mass deviation (ppm)	Isotopic fine structure sulfur
1	DON-Hexitol (e.g.: Mannitol)	4.89	[M+Na]^+^	483.1838	-2.9	
2	DON-S-cysteine* ^ǂ^	5.59	[M+H]^+^	418.1531	-2.9	x
3	DON-S-cysteinyl-glycine* ^ǂ^	6.27	[M+H]^+^	475.1746	-2.2	x
4	DON-glutathione (GSH) * ^ǂ^	7.71	[M+H]^+^	604.2173	-2.4	x
5	DON-di-hexoside	8.19	[M+Na]^+^	643.2211	-2.7	
6	DON**	8.60	[M+Na]^+^	319.1153	-2.2	
7	DON-3-β-D-glucoside** ^ǂ^	9.16	[M+Na]^+^	481.1682	-1.6	
8	“DON-2H”-glutathione*	11.48	[M+H]^+^	602.2014	-2.2	x
9	DON-malonylglucoside*	11.73	[M+Na]^+^	567.1686	-2.6	
10	15-acetyl-DON-3-β-D-glucoside**	13.71	[M+Na]^+^	523.1786	-2.5	

* Annotation based LC-HRMS/MS spectra revealing characteristic fragment ions.

** Identification of compounds based on authentic standard with similar retention time and LC-HRMS/MS spectra (data not shown).

^ǂ^ Biotransformation products have also been putatively identified in a previous study [24].

**Fig 1:**
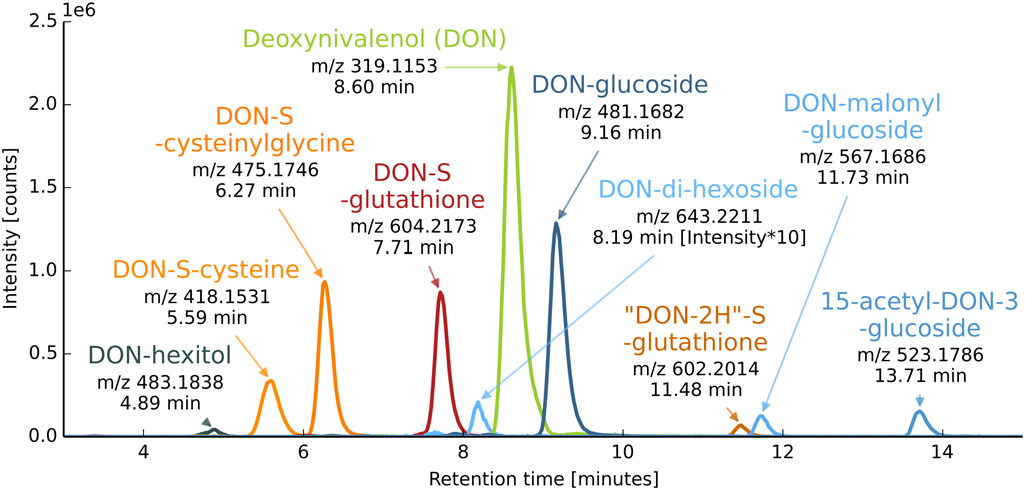
EICs of DON and its corresponding biotransformation products. EICs of accurate mass traces (± 3 ppm) of DON and its corresponding biotransformation products in a wheat sample harvested 96 hours post treatment with 1 mg DON. Due to low abundance, EIC intensities of DON-di-hexoside were multiplied by a factor of 10.

Three sulfur containing metabolites, namely DON-glutathione (GSH), DON-S-cysteinylglycine and DON-S-cysteine had already been putatively identified [24]. Herein, we putatively describe the characterization of the remaining DON biotransformation products. Based on accurate mass measurement and isotopic fine structure, one more GSH conjugate (#8) was detected, in which the glutathione moiety is intact, but the DON moiety is putatively oxidized by the loss of two hydrogen atoms. Thus, this compound was provisionally designated “DON-2H”-glutathione. Another DON derivative most probably constitutes to the toxin being conjugated to a hexitol (#1). Alternatively, this compound may be a derivative formed from D3G by introduction of 2H atoms. The remaining four biotransformation products have been annotated as D3G (#7), DON-malonylglucoside (#9), 15-acetyl-DON-3-glucoside (#10) and a DON-di-hexoside (#5) based on the arguments discussed below. Fig. 1 shows an overlay of EIC of DON and all corresponding biotransformation products in an extract of a DON treated wheat ear (1 mg, 96 h) of the cultivar ‘Remus’.

### Putative structure annotation of newly found DON conjugates

**Fig 2:**
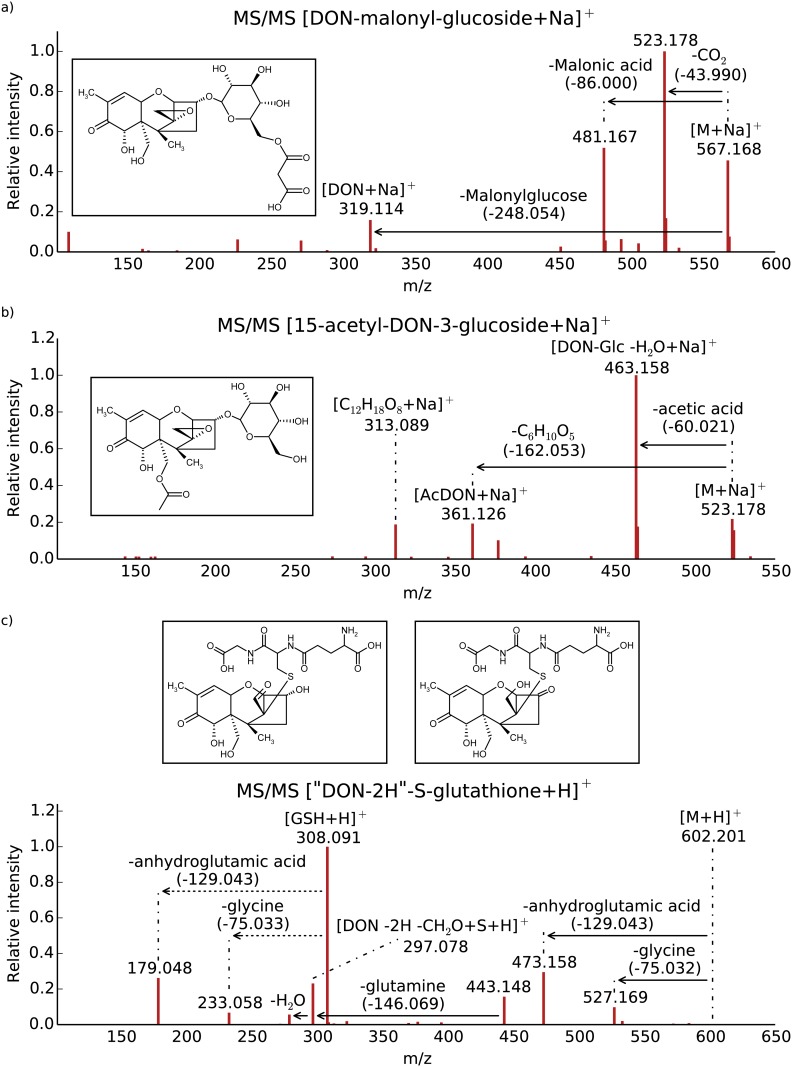
LC-HRMS/MS spectra of the DON biotransformation products. a) DON-malonylglucoside, b) 15-acetyl-DON-3-β-D-glucoside and c) “DON-2H”-S glutathione (GSH), all LC-HRMS/MS spectra are shown with a proposed structure formula.

To confirm the proposed structures of DON-hexitol (#1), “DON-2H”-GSH (#8), DON-malonylglucoside (#9) and 15-acetyl-DON-3-*β-D*-glucoside (#10), LC-HRMS/MS product ion measurements of DON-treated wheat samples were carried out in Collision Induced Dissociation (CID) mode (Fig. 2). Due to low intensities of the precursor ion of #1 *m/z* 483.1824 (DON-hexitol, [M+Na]^+^) no meaningful product ion spectra could be recorded. To verify whether this conjugate possibly consists of reduced DON, conjugated to a glucose moiety, standard 3,7,8,15-tetrahydroscirpene-3-glucoside was measured. This standard showed the same *m/z*, but had a different retention time in the chromatogram and therefore our data support the assumption that DON is conjugated to a hexitol. For the biotransformation product #5 *m/z* 643.2193 (DON-di-hexoside, [M+Na]^+^) also no meaningful product ion spectra could be recorded due to low intensities. Thus, annotation for these two biotransformation products is based on accurate *m/z*, assumed ion species, number of DON derived carbon atoms per conjugate and evaluation of isotopic fine structure.

**DON-malonylglucoside**. Within the full scan mass spectrum, adducts at *m/z* 567.1671 [M+Na]^+^ and *m/z* 589.1287 [M+2Na-H]^+^ have been detected, therefore it was concluded that [M+Na]^+^ is consistent with the sum formula C_24_H_32_O_14_Na (-2.6 ppm mass deviation). Evaluation of the LC-HRMS full scan spectra did not give evidence for mass signals corresponding to ^15^N or ^34^S isotopologs. LC-HRMS/MS measurements of precursor mass *m/z* 567.17 (corresponding to [M+Na]^+^) using 18% relative collision energy revealed mass increments between two signals in the LC-HRMS/MS spectrum corresponding to neutral losses, Δ43.990 u, indicating the loss of carbon dioxide; Δ86.000 u and Δ248.054 u, corresponding to the loss of the malonylglucoside moiety minus water from the intact DON conjugate (Fig. 2a). Moreover, the intact DON molecule was observed (*m/z* 319.114, [M+Na]^+^) in the product ion spectra. These findings are in good agreement with LC-HRMS/MS spectra of plant derived malonylglucoside conjugates such as flavanoids [40]. The conjugate is putatively identified as DON-3-MalGlc since it is assumed that this conjugate is formed from further metabolism of initially formed D3G.

**15-acetyl-DON-3-*β-D*-glucoside**. In the full scan mass spectrum, the mass 523.1773 which corresponds to the [M+Na]^+^ ion of acetyl-DON-glucoside was detected. Subsequent LC-HRMS/MS measurements in the CID mode with 26% relative collision energy (Fig. 2b) revealed the neutral losses of Δ60.021 indicating a loss of an acetyl group and Δ162.053 (C_6_H_10_O_5_) corresponding to the loss of glucose as already described by Dall’Asta *et al*. [39] for D3G. Together with the presence of the fragment at *m/z* 301.126, which corresponds to the sodium adduct of the acetylated DON, these findings suggest that both, the acetyl moiety as well as the glucose molecule are both covalently linked to the DON precursor at different positions. An authentic standard prepared in house by enzymatic in vitro synthesis using recombinant *Escherichia coli* expressed glucosyltransferase and 15-ADON as substrate (manuscript in preparation), for which the same retention time and LC-HRMS/MS spectrum was observed, thus the compound was identified as 15-acetyl-DON-3-*β-D*-glucoside (15ADON3G). Since 15-ADON was not detected in the DON used for plant treatment and none of the other DON-Glc derivatives was observed in acetylated form, we assume that 15ADON3G presumably has been formed from acetylation of D3G.

**“DON-2H”-S-glutathione conjugate**. The mass of the observed intact molecule differed by Δ -2.016 u compared to DON-GSH. Analysis of its isotopic fine structure in LC-HRMS full scan spectra revealed the presence of sulfur within that molecule. LC-HRMS/MS measurements were performed at 18% relative collision energy. Observed fragments indicate the conjugation of an intact glutathione molecule to “DON-2H” (Fig. 2c). Observed neutral losses of Δ75.032 u (glycine), Δ129.043 u (anhydroglutamic acid) are in good agreement with our previous study [24], as well as the fragments of a glutathione moiety described by Levsen *et al*., [41]. The presence of GSH in #8 is further supported by the observation of a fragment to the protonated intact glutathione moiety (*m/z* 308.091) and its corresponding LC-HRMS/MS fragments. Interestingly the LC-HRMS/MS spectrum also showed the S-containing fragment at *m/z* 297.078, but lacked *m/z* 297.133 [DON+H]^+^ indicating that in addition to GSH conjugation DON had been oxidized by the loss of two hydrogen atoms. Glutathione can form a Michael adduct at the C10 atom [42]. An alternative reaction described for glutathione S-transferases is the opening of the epoxide ring [43]. We speculate that after nucleophilic attack of the SH group of glutathione to the C12 atom of DON, the epoxide ring is opened. Subsequently an—OH group is oxidized to an aldehyde or ketone (-2H). This could occur at the primary alcohol newly formed by the epoxide opening (Fig. 2c right) or at the C3-OH of DON (Fig. 2c left). Such a conversion to a keto group by a bacterium has been described [44]. Alternatively, if only the glutathione adduct formation occurs at the double bond at C10, the C8-OH group may be converted into a keto group.

According to the current knowledge on metabolism of xenobiotics, we assume that during phase II metabolism DON is conjugated to endogenous molecules by two major metabolism routes, namely 1) glucosylation and 2) conjugation to the tripeptide glutathione (GSH) as well as the conjugation to a hexitol. These findings are in good agreement with former reports, which delineate conjugation of xenobiotics to glucose and GSH as major detoxification reactions *in planta*. While the detoxification of DON via the conjugation to D3G has already been reported earlier for *Arabidopsis* [19], maize cell suspension cultures [45] and naturally as well as artificially contaminated wheat [20], the conjugation of DON to GSH has only been reported recently [24]. It has been shown, that even the S-methyl adduct already leads to lower inhibition of protein synthesis, it may be assumed that the much bulkier cysteine or glutathione adduct also prevents interaction with the ribosomal target [42]. Our data suggest that the DON-GSH conjugate is stepwise degraded to DON-S-Cys-Gly and DON-S-Cys in wheat. Similar detoxification mechanisms have already been reported for glutathione S-conjugates of the herbicide alachlor in the vacuole of barley [11].

In addition, initially formed D3G is further metabolized to DON-MalGlc and DON-di-hexoside respectively. In agreement with our study, a MalGlc conjugate has already been reported as a detoxification product of 2,4-dichlorophenol in cell-suspension cultures of tobacco [12], and the malonylation of phenolic glucosides has also been reported to be a key reaction in xenobiotic metabolism of *Arabidopsis* [46].

In an earlier study, DON, D3G, DON-di-glucoside and further DON-oligoglycosides were detected in barley based products during beer production after pre-concentration of DON derivatives with immunoaffinity columns and LC-HRMS analysis [21]. In the present study no tri- or higher hexoside conjugates were detected which might be explained by the fact that no sample pre-concentration was employed. Thus, the presence of DON-di-hexoside suggests that further oligoglucosides might be formed in wheat, but due to limited sensitivity were not detected by our approach.

### Mass balance of DON and D3G in wheat

To further assess the metabolic rate of DON in wheat we have estimated a molar mass balance of DON and its main biotransformation product D3G for the investigated wheat samples. For both, DON (*m/z* 297.1334, [M+H]^+^) and D3G (*m/z* 459.1862, [M+H]^+^) matrix calibration was carried out at a concentration range from 1–10 mg L^-1^. First the recovery rate of DON in wheat extracts was determined using five biological replicates harvested directly after treatment with 1 mg DON (≙ 3.38 μmol). On average 3.32 ± 0.07 μmol DON were detected in the samples, corresponding to a recovery rate of 98%. Thus, it can be concluded that DON is not irreversibly bound to wheat matrix within the first few minutes after application (sampling time point 0 h). ‘Remus’ wheat ears treated for 96 hours (n = 5) showed that 65% of the added DON (≙ 2.09 ± 0.21 μmol) were still present with 15% of the applied DON transformed to D3G (≙ 0.50 ± 0.08 μmol). Consequently, all other biotransformation products at most add up to a maximum of approximately 20% (≙ 0.62 ± 0.23 μmol) relative to the DON initially applied to the wheat spikelets. The molar mass balance demonstrates that the detected biotransformation products (including D3G) make up a significant percentage of the metabolized DON and thus both metabolism routes play an important role in the detoxification process of DON. Further detailed studies with respect to the occurrence of the different DON-conjugates in naturally contaminated cereals/wheat will have to be performed to investigate their toxicological relevance.

### Time course kinetics of the formation of DON conjugates in DON treated wheat lines with different genetic background

The kinetics of the formation of the annotated biotransformation products in the investigated set of DON treated wheat lines have been elucidated with the aim to find QTL specific or QTL associated DON conjugation behavior. To this end, the parent wheat lines ‘CM-82036’ (resistant to FHB, harboring the resistance QTL *Fhb1* and *Qfhs.ifa-*5A) and ‘Remus’ (susceptible), and four near isogenic lines (genome 98% identical to ‘Remus’) C1 (+*Fhb1*/+ *Qfhs.ifa-5A*), C2 (+*Fhb1*), C3 (+*Qfhs.ifa-5A*) and C4 (no QTL) were studied. Integrated peak areas of each biotransformation product were normalized to the average EIC peak area observed for the respective biotransformation product in the control aggregate sample to account for changes of the MS sensitivity between different measurement sequences. For the ease of comparability of the respective biotransformation product across the different samples, the chromatographic peak areas were related to the maximum peak area in any of the tested wheat samples. Additionally, box plots were established for each wheat line 96 h after treatment. A non-paired t-test (5% global significance threshold) was performed for the univariate comparison of biotransformation products between wheat lines with and without harboring the resistance QTL *Fhb1*. With the exception of DON-di-hexoside, for which the abundance was too low, time course profiles could be established for every detected biotransformation product. For the mock treated samples none of the EICs showed a peak, confirming the high selectivity of the applied LC-HRMS approach (for raw data of integrated peak areas see S1 Table).

**Fig 3:**
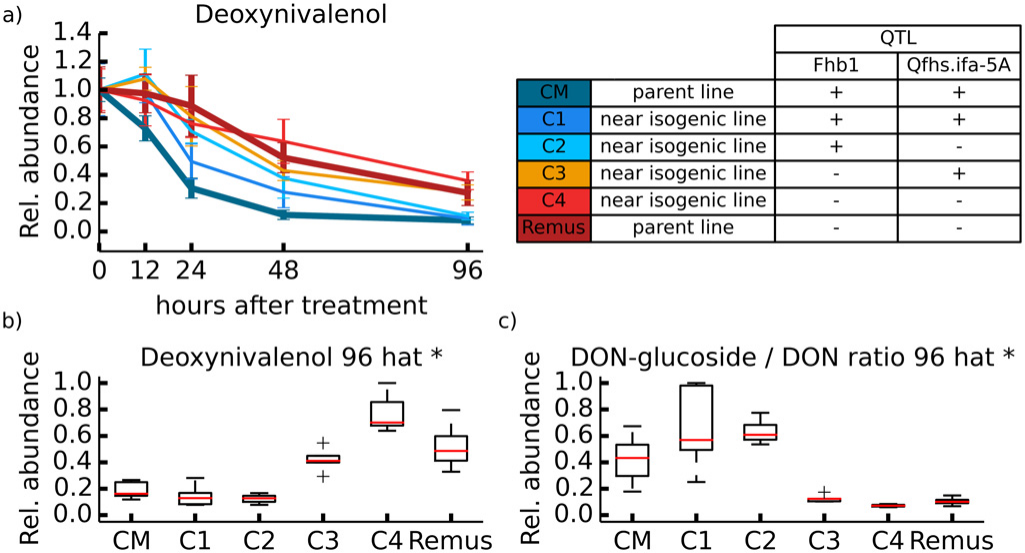
Overview on DON degradation. Time course for the degradation of DON (1 mg) of wheat lines ‘CM-82036’, C1, C2, C3, C4 and ‘Remus’. Wheat ears were sampled 0, 12, 24, 48, and 96 hours after treatment (n = 5 biological replicates per time point and wheat line). a) Degradation rate of DON. b) boxplot of relative concentrations 96 h after DON treatment. c) DON-glucoside/DON ratio 96 h after DON treatment. * Significantly differing DON levels between wheat lines with and without resistance QTL *Fhb1* based on a non-paired t-test (5% global significance threshold).

**Fig 4:**
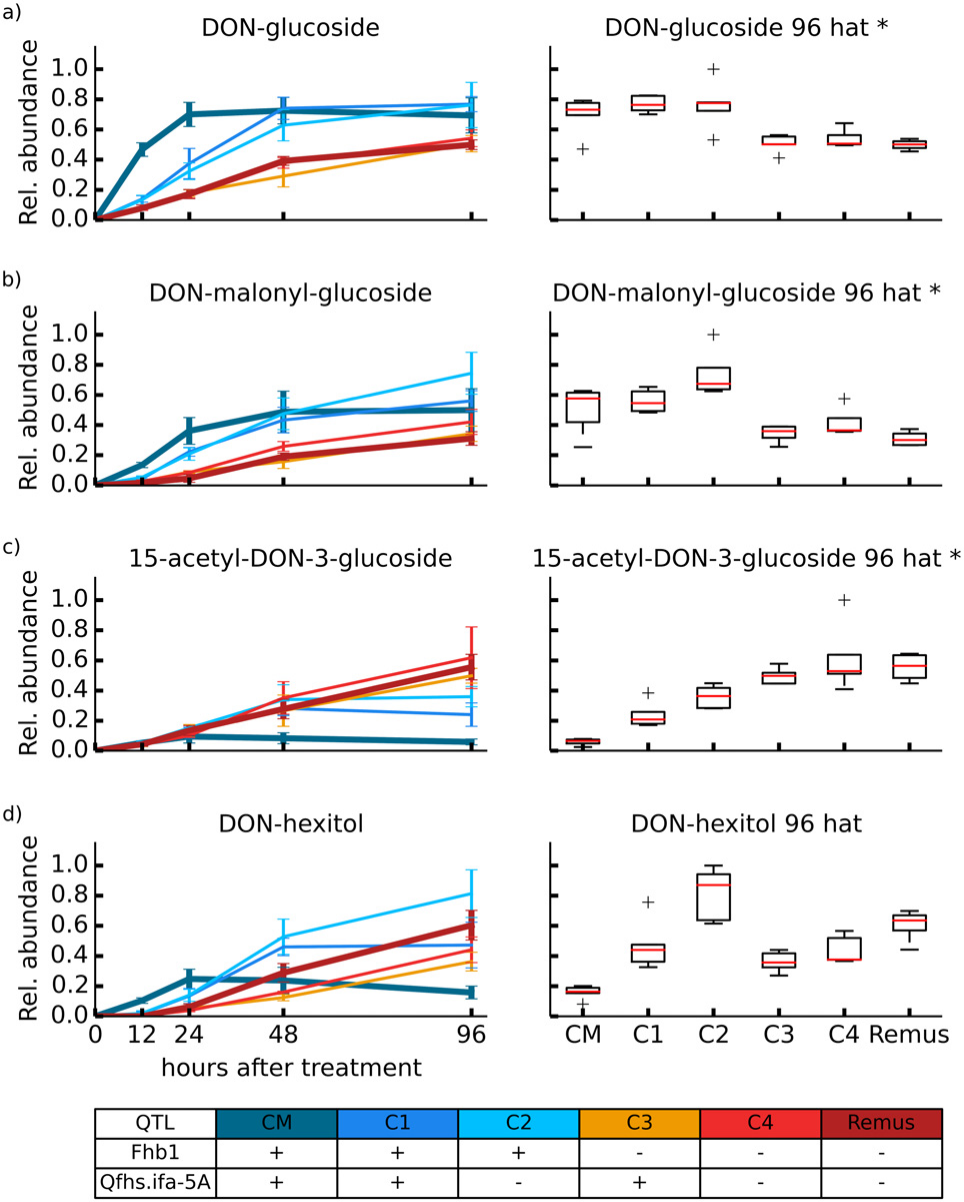
Detoxification of DON via glucosylation/sugar alcohol. Glucose/sugar alcohol related detoxification of DON. Relative formation rates for the biotransformation products DON-glucoside (a), DON-malonyl-glucoside (b), 15-acetyl-DON-3-glucoside (c) and DON-hexitol (d). Additionally, for each biotransformation product boxplots for relative metabolite abundance observed 96 h after DON treatment were generated. * Significantly differing biotransformation product levels between wheat lines with and without resistance QTL *Fhb1* based on a non-paired t-test (5% global significance threshold).

**Fig 5:**
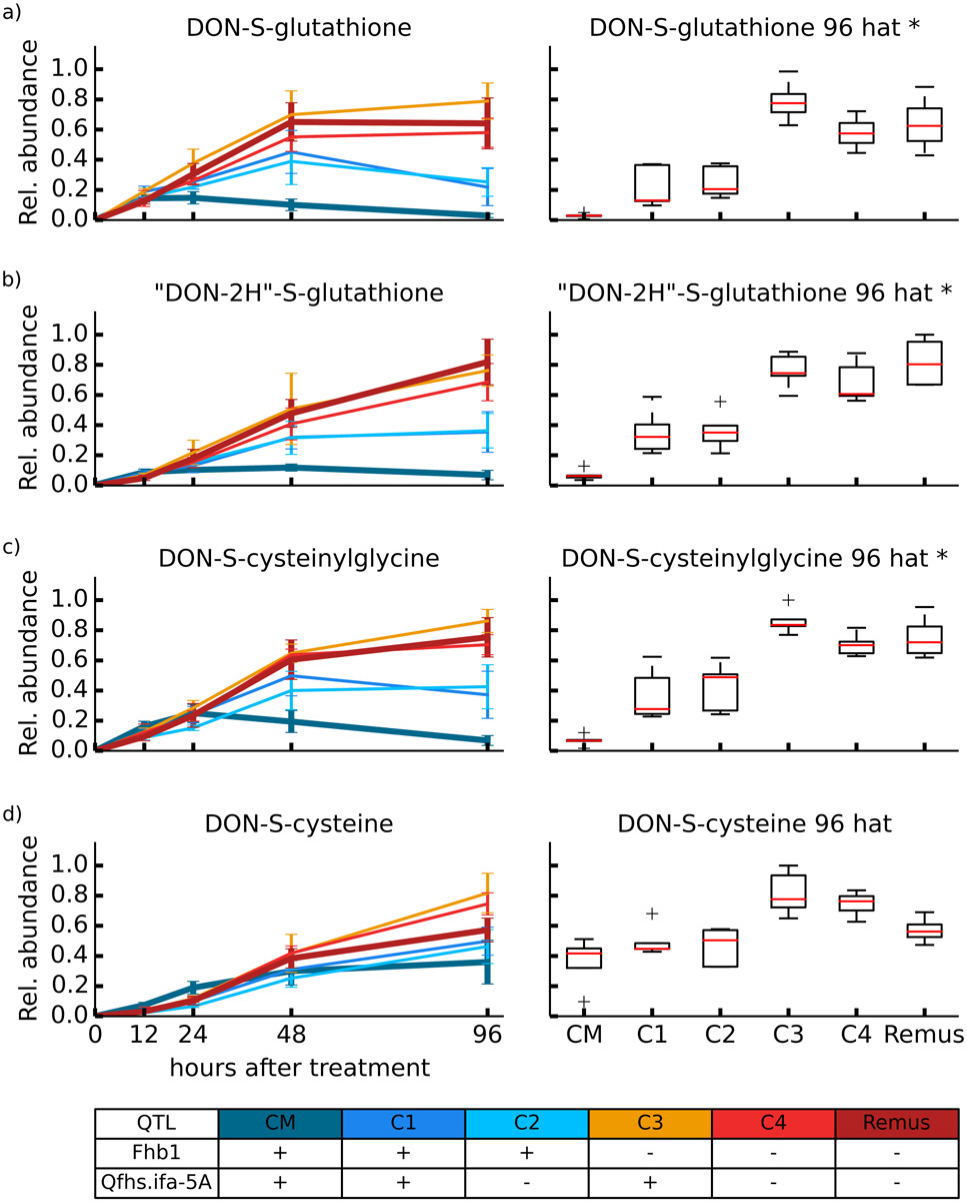
Detoxification of DON via glutathione pathway. Glutathione pathway related detoxification of DON. Relative formation rates for biotransformation products DON-S-glutathione (a) and its related degradation products “DON-2H”-S-glutathione (b), DON-S-cysteinylglycine (c) and DON-S-cysteine (d). Additionally, for each biotransformation product boxplots for relative metabolite abundance observed 96 h after DON treatment were generated. * Significantly differing biotransformation product levels between wheat lines with and without resistance QTL *Fhb1* based on a non-paired t-test (5% global significance threshold).

**Degradation rate of DON**. In this study DON was applied once (time point 0) and consequently DON levels continuously decreased over time in all wheat lines demonstrating that each of the tested wheat lines is capable of metabolizing DON (Fig. 3a). Wheat lines containing *Fhb1* (‘CM-82036’, C1, C2) showed a faster decrease of DON compared to the wheat lines without *Fhb1* (C3, C4, ‘Remus’). The fastest DON metabolic transformation rate of all wheat lines was observed in ‘CM-82036’ indicating the efficient detoxification potential of this cultivar. After 96 h relative concentrations of DON (Fig. 3b) were observed to be significantly higher in wheat lines, which do not harbor the resistance QTL *Fhb1*. In contrast *Fhb1* harboring wheat lines showed significantly lower relative DON concentration levels after 96 h indicating the presence of an efficient mechanism to lower the toxin concentration. As shown in Fig. 3c, D3G/DON ratio under the mentioned test conditions is significantly higher in wheat lines ‘CM-82036’, C1 and C2, all harboring the resistance QTL *Fhb1*. Time course profiles of all biotransformation products (except DON-di-hexoside) of DON were examined with respect to the different combinations of FHB resistant QTL *Fhb1* and *Qfhs.ifa-5A* across the six wheat lines and are illustrated in Figs. 4 and 5 respectively.

**Detoxification of DON via glucosylation/sugar alcohol conjugation**. The resistant cultivar ‘CM-82036’ showed the fastest D3G formation rate of all tested wheat lines (Fig. 4a). The maximum D3G concentration was already reached 24 h after treatment with DON. Also the NILs C1 and C2, both harboring the resistance QTL *Fhb1*, showed higher formation rates of D3G compared to lines without that specific QTL. These findings support the earlier hypothesis that *Fhb1* influences the ability to detoxify DON via glucosylation [28]. In this respect DON-MalGlc (Fig. 4b), which is assumed to be derived from D3G showed a time course similar to D3G. Wheat lines carrying *Fhb1* showed faster formation of DON-MalGlc within 48 h after inoculation, and higher concentration levels 96 h after inoculation compared to those without *Fhb1*. D3G as well as DON-MalGlc showed significantly higher concentrations 96 h after treatment of wheat lines harboring *Fhb1*.

Interestingly, the time course for the formation of 15ADON3G (Fig. 4c) was different compared to that of the other DON-glucose derivatives D3G, DON-MalGlc. Concentration levels of 15ADON3G in C3, C4 and ‘Remus’ were increasing continuously during the observation period and showed significantly higher levels after 96 h than the wheat lines carrying *Fhb1*. This suggests that first 15-ADON is formed, in a competitive reaction, which is less pronounced in lines harboring *Fhb1*, thus, the subsequent formation of 15ADON3G is less favored in these wheat lines. The acetylation of DON is clearly performed by the plant, no ADON was present in the applied DON, and no 15ADON3G was observed in the samples harvested immediately after application with DON.

The kinetics of the putatively annotated DON-hexitol did not show a clear trend between wheat lines harboring *Fhb1* and without. Again the resistant cultivar ‘CM-82036’ showed the highest relative concentration 24 hours after treatment and then continuously decreased whereas all other wheat lines showed continuously increasing formation rates.

**Detoxification of DON via the glutathione pathway**. In contrast to glucosylated DON derivatives, detoxification to DON-GSH and its further degradation products, DON-S-Cys-Gly, DON-S-Cys and “DON-2H”-GSH was more efficient in ‘Remus’, C3 and C4, which all lack the resistance QTL *Fhb1*. Twelve hours after treatment all wheat lines roughly showed the same concentration levels of DON-GSH (Fig. 5a), while at later time points the cultivars harboring the QTL *Fhb1* showed significantly lower concentration levels for DON-GSH, DON-S-Cys-Gly and “DON-2H”-GSH. Only DON-S-Cys showed rising levels throughout the whole observation period indicating that this metabolite is being formed from DON-GSH via DON-S-Cys-Gly. One reason for the different kinetics between glucose- related and GSH- related metabolism might be that in the *Fhb1* harboring wheat lines, the formation of D3G is more efficient and thus the DON for the competing formation of DON-GSH and related derivatives is less available in these wheat lines. An apparent problem with this interpretation is that there is still a huge excess of unmetabolized DON detected in the presented experiment. Yet, this does not take into account that the intracellular concentration of DON may be much lower due to the action of drug efflux pumps, which are highly induced according to gene expression studies of DON treated barley [47], DON treated wheat [48] or *F*. *graminearum* inoculated wheat [49,50]. The pool of intracellular DON may therefore be limiting for the competing enzymes, so that DON can be metabolized preferentially into one or the other major pathway.

In this study DON was only applied as a single dose to all wheat lines and thus the resulting conclusions were made based on the described conditions. We conclude that all wheat lines under investigation have both, glucosylation-related as well as the glutathione pathway for the detoxification of DON. The detoxification of DON is faster in *Fhb1* harboring wheat lines, which can be mainly attributed to the formation of D3G and DON-MalGlc. The time course profiles as well as the relative concentrations 96 h after treatment show higher glucoside related biotransformation (D3G, DON-MalGlc) in *Fhb1* harboring wheat lines. The relative concentrations of GSH related metabolites (except DON-S-Cys) are significantly higher 96 h after treatment in wheat lines lacking *Fhb1*. In many plant-pathogen systems the dynamics of the plant resistance reaction has been described to be decisive for the outcome “diseased or resistant” [51]. As our findings clearly demonstrate that the presence of the QTL *Fhb1* was correlated with an increased metabolism of DON, it can be concluded that the speed of DON detoxification seems to be a decisive factor for resistance towards FHB enhancing the efficient glucosylation of DON.

Moreover, as our experiment employed treatment with pure toxin, the observed metabolic response *in planta* can be clearly attributed to DON only. It shall be noted however that the time course profiles and D3G/DON ratios obtained for the single dose DON treatment applied in this study, cannot directly be compared to the situation found during natural *F*. *graminearum* infection. Compared to single dose toxin treatment, continuous DON production as well as the release of additional fungal low molecular weight effectors and proteins, modulates the defense response under natural infection conditions. Transcription of UDP-glucosyltransferases and glutathione-S-transferases have been shown to be highly DON inducible [47,50]. Whether these transcripts can be translated into active detoxification enzymes depends on the intracellular concentration of DON. While protein biosynthesis may be completely blocked at the site of DON application, lower levels due to diffusion should allow induction of the detoxification enzymes in neighboring tissue. In the study of Lemmens *et al*. two adjacent spikelets were treated with DON, and the whole ear was extracted after ripening (21 days). As both, infected as well as non-infected spikelets of DON treated wheat ears were included by Lemmens *et al*., an efficient detoxification of DON could be expected at sites of relatively low toxin concentration (distant from the infected spikelets). The different rates of DON formation of the tested wheat lines can therefore explain the large differences in D3G/DON ratio found by Lemmens *et al*. In contrast, no significant difference in the D3G/DON ratio was found in inoculated spikelets and rachis between NILs differing in *Fhb1* 72 hours post inoculation (Gunnaiah *et al*., 2012). This might be due to high local DON concentrations preventing an efficient DON metabolism directly in the infected tissue. In conclusion, both studies have an entirely different study design, with totally different toxico-kinetics and—dynamics (e.g. single application versus continuous DON production by the fungus, different tissue as well as time point of sampling), which could result in the described different findings. Another complication explaining seemingly contradictory results is that the advancing fungus, which overwhelms the initially inoculated spikelet regardless of the genotype, has the ability to efficiently reactivate D3G initially detoxified and stored in the vacuole with powerful β-glucosidase (data not shown). The capability of enzymatic hydrolysis of D3G has also been demonstrated for cellulase from *Trichoderma* and cellobiase from *Aspergillus* species [52].

## Conclusion

In the present study, which aimed to study the metabolism of DON in wheat, four DON biotransformation products have been assigned to glucose- and another four to GSH-related DON detoxification pathways. Comparison of a set of six wheat lines, which carry different combinations of the two major resistance QTLs (*Fhb1* and Qfhs.ifa-5A) against FHB, revealed a faster D3G and DON-MalGlc formation in *Fhb1* lines. DON belongs to the group of trichothecene mycotoxins and is well known as a major virulence factor of *F*. *graminearum* most probably by inhibiting the biosynthesis of defense related proteins. Under our conditions, DON detoxification in wheat is clearly correlated with a major QTL for FHB resistance, *Fhb1*, and results in different formation rates of the respective DON biotransformation products.

Due to the treatment with pure toxin at the beginning of flowering in this study, all observed QTL specific effects can be clearly attributed to the presence of DON. However, these effects are not always evident in *Fusarium* infection experiments, which might also be attributed to the different experimental designs in the studies presented by Gunnaiah et al. [29] and Lemmens et al. [28] respectively. The occurrence of the detected conjugates in naturally *F*. *graminearum* infected cereals and their role as potential masked mycotoxins warrant further investigation in the future.

## Supporting Information

S1 Table Peak areas of DON biotransformation products used to establish the time courses, which are presented in this study. (TXT)
